# Fractionated but not single dose radiation releases key signals of in situ tumor vaccination

**DOI:** 10.1186/2051-1426-2-S3-P164

**Published:** 2014-11-06

**Authors:** Claire Vanpouille-Box, Molykutty Aryankalayil, Karsten Pilones, Silvia Formenti, C Norman Coleman, Sandra Demaria

**Affiliations:** 1 New York University School of Medicine, New York, NY, USA; 2 National Cancer Institute, Bethesda, MD, USA

## 

The balance between pro-inflammatory and immunosuppressive signals in the tumor microenvironment dictates the responsiveness of the immune system. Local radiotherapy (RT) has the potential to switch this balance in favor of anti-tumor immunity by promoting cross-priming of anti-tumor T cells thus generating an individualized *in situ *vaccine. We have previously shown that the dose and fractionation employed modulate RT ability to synergize with immunotherapy. Indeed, in two tumor models, generation of an *in situ *vaccine synergistic with anti-CTLA-4 treatment was achieved by irradiation of the tumor with 3 fractions of 8 Gy (8Gyx3) but not by a single 20 Gy dose (20Gyx1) (Dewan *et al*., Clin Cancer Res 2009).

To understand the mechanisms underlying the different outcome obtained with fractionated (3x8Gy) versus single dose (20Gyx1) RT, TSA tumors growing in syngeneic immunocompetent BALB/c mice were harvested at 4, 24 and 48 hrs post-RT for analysis of purified RNA by microarray for gene expression or infiltrating immune cells by flow cytometry. Expression of key immune genes in TSA cells irradiated *in vitro *was assessed by qPCR.

Over 100 immune response genes were differentially expressed in irradiated tumors by 8Gyx3 but not 20Gyx1, with a dominant type I interferon (IFN) response at 4 and 24 hours, which was confirmed by qRT-PCR. CD8a+ dendritic cells (DC), which are the subset of DC cross-presenting tumor cell-derived antigens, showed a significant upregulation of activation markers CD86, CD40 and CD70 at 48 hours following 8Gyx3 but not 20 Gyx1. Importantly, the *in vitro *setting (devoid of an immune infiltrate) demonstrated expression of IFNb and downstream immune genes, including chemokines CXCL9, CXCL10 and CXCL11 by TSA cells irradiated with 8Gyx3 but not 20Gyx1.

Data indicate that fractionated RT can mimic, at least in part, a viral infection and activate canonical defense pathways in neoplastic epithelial cells with induction of type-I IFN. *In vivo *this leads to activation of DC cross-presenting tumor antigens, suggesting that the quality of fractionated-RT generates the key "ingredients" of an *in situ *tumor vaccine. Further studies to identify the molecular mechanisms of RT-induced tumor vaccination and their modulation by different RT regimens are critical to the rational design of clinical trials testing RT combinations with immunotherapy.

